# Developmental Skills Moderate the Association Between Core Autism Features and Adaptive Behaviour in Early Childhood

**DOI:** 10.1007/s10803-023-05932-9

**Published:** 2023-05-04

**Authors:** Daniel Berends, Catherine A. Bent, Giacomo Vivanti, Cheryl Dissanayake, Kristelle Hudry

**Affiliations:** 1 Department of Psychology Counselling and Therapy, School of Psychology and Public Health, La Trobe University, Melbourne, Australia; 2 A.J.Drexel Autism Institute, Drexel University, Philadelphia, USA; 3 Olga Tennison Autism Research Centre, School of Psychology and Public Health, La Trobe University, Melbourne, Australia

**Keywords:** Core Autism Features, Associated Abilities, Developmental Skills, Cognitive Skills, Intellectual Disability, Adaptive Behaviour

## Abstract

**Purpose:**

While research indicates that both the core features of autism and associated developmental skills influence adaptive behaviour outcomes, results to date suggest greater influence of the latter than the former, and little attention has been given to how the interaction of both together might impact functional disability. Seeking to expand understanding of associations between young children’s core social autism features, developmental skills, and functional ability/disability, we specifically tested whether early developmental skills might have a moderating effect on the association between early social features and subsequent functional disability.

**Methods:**

Data from 162 preschool children were available for this study. These included time-1 measures of social autism features (ADOS-Social Affect score) and developmental skills (MSEL-Developmental Quotient; DQ), and a measure of functional ability/disability (VABS-Adaptive Behaviour Composite; ABC) available at follow-up 1-year later (time-2).

**Results:**

Time-1 ADOS-SA and MSEL-DQ scores were concurrently associated with one another, and both prospectively associated with time-2 VABS-ABC scores. Examination of partial correlations (i.e., controlling for MSEL-DQ) demonstrated that the association of time-1 ADOS-SA and time-2 VABS-ABC was accounted for by shared variance with DQ. Formal moderation analysis returned a non-significant overall interaction term, but showed a lower-bound region of significance whereby the association of time-1 ADOS-SA with time-2 VABS-ABC was significant for children with baseline DQ ≤ 48.33.

**Conclusion:**

Our results add to a body of empirical evidence consistent with an understanding of the needs of and resources available to autistic people through a ‘cognitive compensation’ lens.

## Introduction

Autism Spectrum Disorder (ASD; hereafter *autism*) is a neurodevelopmental condition characterised by social attention and communication differences, as well as the presence of restricted/repetitive behaviours and interests (American Psychiatric Association, 2022). Autism is otherwise marked by substantial heterogeneity, with variation in the presentation of core features and also in domains of broader developmental/cognitive skills and adaptive behaviour. That is, in addition to the highly variable core features, cognitive abilities vary from well-below to average to well-above average (Georgiades et al., 2013; Kim et al., 2016). Further, there is evidence that presentation of core features and cognitive abilities also varies within individuals over time (Whitehouse et al., 2017).

Intellectual Disability (ID) is common in autism, defined as < 70 points on a standardized Intelligence Quotient (IQ) test and within the lower 2nd percentile on a standardized test of adaptive behaviour (American Psychiatric Association, 2022). Estimates of ID in autistic samples range widely, from 33 to 70% (Fombonne, 2003; La Malfa et al., 2004; Maenner et al., 2020), and compared to estimates of 1 to 2% in the general population (Elsabbagh et al., 2012). Until recently, the observed association of autism with ID has been explained in terms of *comorbidity*; the independent co-occurrence of two or more distinct conditions in a given individual. However, recent theorisation and empirical research efforts have sought potential causal explanations for this markedly increased co-occurrence of core autism features and ID.

### Associations Between Core Autism Features and Developmental Skills

Empirical research has demonstrated an association between the early core features of autism and gains in developmental skills. Vivanti et al. (2013) investigated the association between autism features and developmental skills in two independent samples. First, among 23 children receiving the Early Start Denver Model (ESDM), where those with more early core autism features had lower concurrent developmental skills, and also subsequently made fewer developmental gains at 1-year follow-up. Second, among a sample of 60 community-referred infants and toddlers showing early signs of autism was the same pattern of results; more early core autism features correlated with lower concurrent developmental skills, but also with subsequently slower developmental growth across a 2-year follow-up period.

These findings have been interpreted as evidence for a potential causal link between early autism features and later developmental skills, thereby explaining the observed common co-occurrence of ID in autism. The social learning theory of autism suggests autistic features impact engagement with social stimuli necessary for typical early neuro-cognitive development (Dawson et al., 2004; Vivanti et al., 2013). That is, decreased engagement with social stimuli—a hallmark feature of early autism (Annaz et al., 2012; Mundy, 2018; Perlman et al., 2011)—impacts broader developmental skills usually acquired through social learning (Karmiloff-Smith, 2009).

While Vivanti et al. (2013) considered unidirectional associations only—the relationship between early autism features and later developmental skills—Karmiloff-Smith’s *neuroconstructivist* approach proposes development as a dynamic process in which any impact of neuro-developmental conditions (such as autism) on cognitive development, is unlikely to be *unidirectional* (Karmiloff-Smith et al., 2003). Indeed, we have recently expanded upon the line of inquiry begun in Vivanti et al.’s (2013) study, testing and showing evidence for the *reverse* directional effect in an expanded cohort similar to their preschool-aged group. Drawing on data from 155 children assessed at two timepoints—associated with participation in the same service, and including some of the same children as sampled by Vivanti et al. (2013)—we found evidence for bidirectional cross-lagged associations—between early core autism features and subsequent developmental skills, *and* also between early developmental skills and subsequent core autism features. Further more we found cross-lagged effects to be *stronger* in this latter direction—from earlier developmental skills to later autism features—than in the former (McGowan et al., 2022).

These findings provide empirical support for an alternative theory of the potential causal link between core features of autism and developmental skills; that the developmental skills may be drawn upon to compensate for difficulties otherwise experienced as a function of autism, consistent with *neuroconstructivist theory* (Karmiloff-Smith, 2009). This is also consistent with the notion of *cognitive compensation* (Livingston & Happé, 2017); the understanding that autism originates from innate cognitive differences (Annaz et al., 2012; Perlman et al., 2011) and that autistic individuals may draw upon different cognitive processes to those typically used, to compensate for these differences, thereby moderating the overt presentation of their autism features (Livingston et al., 2018; Ullman & Pullman, 2015).

### Adaptive Behaviour Outcomes in Autism

Real-world functional outcomes and disability can be operationalised in terms of adaptive behaviour—the performance of daily activities required for personal and interpersonal function including communication, socialization, motor, and daily living skills (Sparrow et al., 1984). In a study of 41 autistic children followed across a 20-year period, Farley et al. (2009) found early adaptive behaviour to be a key indicator of later functional outcomes such as gaining independence, maintaining supportive relationships, and better physical and mental health. Perry et al. (2009) assessed concurrent associations of core autism features with domains of adaptive behaviour in 290 autistic children, finding small to moderate association specifically with social and daily living skill domains. They also compared subgroups of 28 autistic and non-autistic children (matched for developmental skills) and found lower adaptive behaviour (specifically in socialisation and communication domains) in the autism group, suggesting autism was associated with adaptive behaviour in these domains independently of developmental skills. Kanne et al. (2010) conducted an investigation with a larger sample of 1,089 autistic children and adolescents aged 4–17 years, also finding small to moderate concurrent associations between global measures of each of autism and developmental ability with global scores of adaptive behaviour. Paul, Loomis, and Chawarska (2011) found a similar pattern of concurrent association in a sample of 54 autistic toddlers.

Developmental skills, core autism features and adaptive behaviour have been shown to be associated with one another in autistic samples. Liss et al. (2001) found that the extent of core autism features in 49 autistic 9-year-olds predicted adaptive behaviour in those with age-appropriate vs. delayed developmental skills (operationalised as IQ above vs. below 80), with IQ more strongly predictive of concurrent adaptive behaviour than autism features in both groups. Additionally, the aforementioned studies found evidence of association of developmental skills (again operationalised in terms of IQ) with overall adaptive behaviour in a large sample of autistic children (Kanne et al., 2010) and a sample of autistic toddlers (Paul et al., 2011). In another sample of autistic toddlers, Ray-Subramanian, Huai, and Weismer (2011) found that adaptive behaviour in the daily living skills domain was correlated with core autism features, and also, that developmental skills were associated with concurrent adaptive behaviour over and above the association with autism features across all domains.

Flanagan et al. (2015) found a longitudinal association between developmental skills and adaptive behaviour among 369 autistic children assessed between two and six years of age. Notably, children with developmental skills in the average or below average range demonstrated improvements in their adaptive behaviour over the follow up period, while those with scores well below-average showed limited to no improvement in adaptive behaviour, or indeed, worsening trajectories. Together these studies show the impact of developmental skills on adaptive behaviour in diverse autistic samples.

Research to date considering adaptive behaviour outcomes in autism has examined the associative effect of one or both of core autism features and/or developmental skills, but has not considered these simultaneously in order to disentangle potential overlapping effects. While the aforementioned studies have found associations between autism features and adaptive behaviour outcomes, adaptive behaviour has tended to be more strongly associated with developmental skills (often operationalised as IQ; Kanne et al., 2010; Paul et al., 2011; Perry et al., 2009). The question of how core autism features and developmental skills might *interact* to predict adaptive behaviour outcomes remains unanswered.

### The Current Study

We sought to expand understanding of the association of core social autism features, developmental skills, and adaptive behaviour in early childhood, testing the predictive association of early social features and developmental skills for adaptive behaviour outcomes across a roughly one-year follow-up period, and the potential moderating effect of early developmental skills on the association between social autism features and adaptive behaviour outcomes. We addressed this question as an extension of our own previous work, drawing on data from the same cohort reported by McGowan et al. (2022), with the novel inclusion of a measure of adaptive behaviour as the key outcome of interest.

We accessed data from 162 children with measures of social autism features and developmental skills available at an initial assessment (hereafter, time-1) and a measure of adaptive behaviour available at follow-up (hereafter, time-2). We hypothesised that autism features and developmental skills would be correlated concurrently, and that both would be correlated prospectively with adaptive behaviour. Furthermore, we hypothesised that the association between earlier autism features and later adaptive behaviour would be moderated by developmental skills, such that autism features would have a stronger association with later adaptive behaviour at lower levels of developmental skills, and weaker association with later adaptive behaviour at higher levels of developmental skills.

## Method

### Participants and Procedure

This study drew on data collected from autistic children enrolled between 2010 and 2018 at a university-affiliated community child-care centre offering Group-based Early Start Denver Model (G-ESDM; Vivanti et al., 2017). This included data for the same 23 pre-school aged children in Vivanti et al.’s study (2013) and 155 children included in our recent analysis (McGowan et al., 2022). Children were assessed both at entry to the service (time-1) and again at follow-up (time-2) after approximately 1-year of support or at exit from the service (depending on duration of enrolment and requirements of other studies/evaluation in which children were participating; follow-up intervals ranged from 3 to 44 months; *M* = 13.5, *SD* = 6.6). Among a cohort of 221 children, the final sample for the current study comprised 162 autistic children for whom complete data were available on key measures of interest. Parents provided written informed consent for their children’s research participation.

Participant demographic characteristics are summarised in Table 1. Children were aged between 13 and 63 months at time-1. Most were male, a minority subgroup were from multiplex families, and almost half had experienced parent-reported developmental regression. Due to substantial variation in follow-up interval, we assessed this as a potential covariate. Multiplex status and parent-reported regression history were also considered as potential covariates due to previous research suggesting these to be associated with developmental skill trajectories in autism (Berends et al., 2019; Gadow et al., 2017).

**Table 1:** Demographic Characteristics of Retained and Excluded Subgroups

	Retained Subgroup *n* = 162	Excluded Subgroup *n* = 59	Significance Test
Male; *n*(%)	123 (75.9%)	50 (86.2%)	*χ*^*2*^ = 2.26, *p* = .132
Age in Months			
Time-1 *M*(SD) Range	33.07 (10.09) 13.50–63.54	39.70 (11.40) 18.96–61.57	*t*(210) = 3.88, *p* < .001
Time-2 *M*(SD) Range	46.79 (11.09) 24.28–78.26	52.46 (10.79) 32.13–71.33	*t*(201) = 2.77, *p* = .006
Multiplex/Simplex status; *n* (%)			
Sibling with ASD	40 (24.7%)	18 (46.2%)	
Siblings without ASD	64 (39.5%)	15 (38.5%)	*χ*^2^ = 4.19, *p* = .041
Only-child	35 (21.6%)	5 (12.8%)	
Developmental Regression; *n* (%)	75 (46.3%)	22 (68.7%)	*χ*^2^ = 3.08, *p* = .380
Follow-up in Months *M*(SD)	13.72 (6.93)	13.19 (5.16)	*t*(195) = -0.43, *p* = .669

*Note*: Percentages for excluded group do not equate to 100% due to missing data

Comparative analyses were conducted between the subgroup of children included in this study and those from the full cohort necessarily excluded here due to missing data on key variables (shown in Table 1). There were no significant differences on key measures of interest of social autism features (*t*(204) = .37,* p* = .711), developmental skills (*t*(70.07) = .01, *p* = .989) or adaptive behaviour (*t*(172) = 1.86, *p* = .065). The retained subgroup of children were significantly younger and more often from multiplex families than those excluded, most likely due to cohort effects linked to changes in enrolment practices of the child-care centre (i.e., children enrolling into intervention services at younger ages from 2014 onwards).

### Design and Measures

Data accessed for this study included direct assessment measures of core autism features and developmental ability collected at time-1, and a parent-report measure of adaptive behaviour collected at both time-1 and time-2.

The Autism Diagnostic Observation Schedule (ADOS; Lord, 2012; Lord et al., 2000) is a measure of behavioural autism features, with one of five modules administered selected based on participant age and verbal capacity (here, Toddler Module or Modules 1, 2 or 3). Given data collection spanned both the ADOS original and 2nd editions, yielding slightly different scores, algorithm scores consistent with the ADOS-2 were calculated following Hus, Gotham and Lord (2014). ADOS Social Affect (SA) domain scores were used for the current analysis (ranging plausibly from 1 to 22, with higher scores indicating greater level of autism features) given our focus on the association between social autism features and developmental skills with adaptive behaviour outcomes (and that restricted/repetitive features were not previously found to be associated with developmental skills in this cohort; McGowan et al., 2022).

The Mullen Scales of Early Learning (MSEL; Mullen, 1995) is a standardised measure of developmental skills for children aged from birth to 68 months, covering four key subdomains: visual reception, fine motor, and receptive and expressive language skills. An overall Developmental Quotient (DQ) was computed as the mean of subdomain age-equivalent (AE) scores / chronological age x 100 such that DQ = 100 indicates skills broadly on-par with a child’s age-related cognitive level (Cho et al., 2022) and lower scores suggest increasing developmental delay/disability.

The Vineland Adaptive Behaviour Scales—2nd edition (VABS; Sparrow et al., 2005) is a frequently utilised measure of adaptive behaviour, yielding a global Adaptive Behaviour Composite (ABC) Standard Score (population *M* = 100; *SD* = 15) summarising across Communication, Daily-Living, Social, and Motor Skill domains. Higher ABC scores indicate greater levels of adaptive behaviour against age-related expectations, here based on parent report of the child’s everyday functional skills.

### Analysis Plan

Following initial confirmation of associations between time-1 ADOS-SA and MSEL DQ and time-2 VABS ABC (and tests of association with child demographic characteristics as potential covariates), regression analysis was planned to test hypotheses regarding the potential predictive main effects of time-1 ADOS-SA and MSEL DQ on time-2 VABS ABC, and the moderating influence of time-1 MSEL DQ on the predictive association of time-1 ADOS-SA on time-2 VABS ABC.

## Results

### Preliminary Data Handling and Sample Characterisation

Data analysis was conducted using SPSS-24 with the PROCESS macro. Datapoints ± 3 standard deviations from the mean—one each for time-1 ADOS-SA and MSEL DQ—were considered outliers and handled by reassignment of a score one point greater/lesser than the nearest non-outlier (Tabachnick & Fidell, 2013). Assumptions of normality and homoscedasticity for regression were met.

Table 2 shows further sample characterisation on key measures of interest. The children demonstrated substantial variation across all measures, with ADOS-SA, MSEL DQ and VABS ABC scores spanning almost the total possible ranges. Group mean scores for each measure were well within the clinical range, as would be expected for a sample of young autistic children who were accessing early learning services. That is, notwithstanding substantial individual variability, the sample average ADOS-SA score suggested clear signs of autism, and sample average time-1 MSEL DQ and time-2 VABS ABC scores were well below the average ranges as indexed against child chronological age.

**Table 2:** Summary of Children’s Scores on Key Measures of Interest

Measure	*n*	*M* (*SD*)	Range
Time-1 ADOS-SA	162	13.93 (4.32)	3–22
Time-1 MSEL DQ	162	62.71 (24.49)	17.44–141
Time-2 VABS ABC	162	76.41 (15.35)	43–121

*Note*: ADOS-SA = Autism Diagnostic Observation Schedule Social Affect algorithm total score, MSEL DQ = Mullen Scales of Early Learning Developmental Quotient, VABS ABC = Vineland Adaptive Behaviour Scales Adaptive Behaviour Composite

### Associations Between Key Measures

Table 3 shows associations of potential covariates—child sex, multiplex/simplex status, developmental regression history and follow-up period—with key measures of interest. Only one significant effect was evident: for child sex and time-1 MSEL DQ.

**Table 3:** T-tests and Pearson’s Correlations Between Key Measures and Potential Covariates

	Time-1 ADOS-SA	Time-1 MSEL DQ	Time-2 VABS ABC
Child Sex	*t*(158) = 1.45, *p* = .148	*t*(158) = -2.31, *p* = .022	*t*(158) = -1.41, *p* = .160
Developmental Regression	*r* = .05, *p* = .566	*r* = -.12, *p* = .218	*r* = -.09, *p* = .364
Multiplex/Simplex status	*r* = -.02, *p* = .775	*r* = .02, *p* = .795	*r* = .00, *p* = .999
Follow-up period	*r* = .04, *p* = .590	*r* = .02, *p* = .758	*r* = -.13, *p* = .094

*Note*: ADOS SA = Autism Diagnostic Observation Schedule Social Affect total algorithm score, MSEL DQ = Mullen Scales of Early Learning Developmental Quotient, VABS ABC = Vineland Adaptive Behaviour Scales Adaptive Behaviour Composite, Child sex coded 0 = male, 1 = female

Table 4 shows the Pearson’s correlations between each of time-1 ADOS SA and MSEL DQ and time-2 VABS ABC scores, which were all moderately to strongly correlated, as expected. Also in Table 4 are the partial correlations computed between each of the two independent variables—time-1 ADOS-SA and MSEL DQ—with the dependent variable—time-2 VABS ABC—whilst controlling for shared variance of the other independent variable. The association between time-1 MSEL DQ and time-2 VABS ABC remained significant when controlling for time-1 ADOS-SA, but there was no longer a statistically significant association between time-1 ADOS-SA and time-2 VABS ABC when controlling for time-1 MSEL DQ.

**Table 4:** Associations between Key Measures (simple correlations in lower left and partial correlations controlling for alternate independent variable in upper right quadrant)

Measure	Time-1 ADOS SA	Time-1 MSEL DQ	Time-2 VABS ABC
Time-1 ADOS SA	-	N/A	*r* = -.10, *p* = .204
Time-1 MSEL DQ	*r* = -.59, *p* = < 0.001	-	*r* = -.62, *p* < .001
Time-2 VABS ABC	*r* = -.48, *p* = < 0.001	*r* = .73, *p* < .001	-

*Note*: ADOS SA = Autism Diagnostic Observation Schedule Social Affect total algorithm score, MSEL DQ = Mullen Scales of Early Learning Developmental Quotient, VABS ABC = Vineland Adaptive Behaviour Scales Adaptive Behaviour Composite

### Moderating Effect of Developmental Skills

**Fig. 1:**
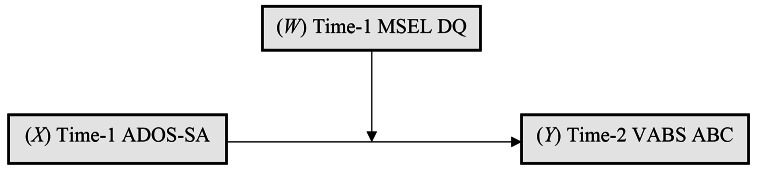
Hypothesised moderating effect of time-1 DQ on the association of time-1 ADOS-SA on time-2 ABC

Regression analysis was conducted using the SPSS PROCESS macro to test the hypothesised moderation represented in Fig. 1. Model estimation parameters were based on ordinary least squares regression with bootstrapped confidence intervals (5000 samples) using time-1 ADOS-SA as the primary predictor of time-2 VABS ABC, and potentially moderated by time-1 MSEL DQ. As the inclusion of child sex (covariate associated with time-1 MSEL DQ) was not statistically significant in the regression model (*p* = .920), it was omitted from the final model presented here.

The overall regression model was statistically significant, *R*^*2*^ = 0.54, *MSE* = 110.71, *F*(1,158) = 61.53, *p* < .001, *β* = 0.73. Table 5 presents the main effects for both time-1 ADOS-SA and MSEL DQ as predictors of time-2 VABS ABC, and the interaction term as a test of potential moderation. Both main effects were significant, whereas the interaction term was not significant with 95%CI including but not crossing the null.

**Table 5:** Main Effects and Interaction Term within Regression Analysis

Model	*b*	*SE*	* t*	* p*	*95%*CI
Constant	65.79	8.66	7.96	< .001	48.68–82.90
Time-1ADOS-SA	-1.18	0.57	-2.09	.038	-2.30 – -0.07
Time-1 MSEL DQ	0.26	0.10	2.57	.011	0.06–0.47
ADOS-SA * MSEL DQ	0.01	0.01	1.71	.089	0.00–0.03

*Note*: ADOS-SA = Autism Diagnostic Observation Schedule Social Affect total algorithm score, MSEL DQ = Mullen Scales of Early Learning Developmental Quotient

**Fig. 2:**
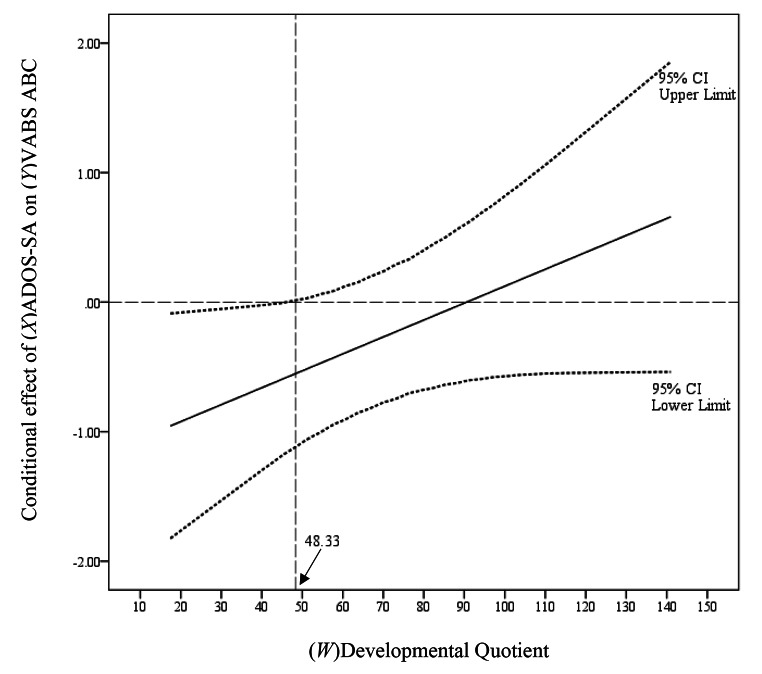
Lower-bound region of significance for conditional effect of ADOS-SA on VABS ABC as a function of MSEL DQ

The stated hypothesis regarding a potential moderation effect of developmental ability was assessed using the Johnson-Neyman approach (Muñoz & Gónzalez, 2017). As shown in Fig. 2, this identified a region of significance suggesting that the prospective association of time-1 ADOS-SA with time-2 VABS ABC was significant for children whose time-1 MSEL DQ score was ≤ 48.33. Conversely, for children with time-1 MSEL DQ > 48.33 there was no significant association of time-1 ADOS-SA on time-2 VABS ABC. We also tested the inverse moderation effect (not shown here), finding no region of significance where association between time-1 MSEL DQ and time-2 VABS ABC was moderated by time-1 ADOS SA.

## Discussion

The aim of this study was to expand on previous investigation of the association between core social autism features and developmental skills in early childhood by introducing outcome measurement of adaptive behaviour and testing a specified moderation effect of developmental skills. As hypothesised, early social autism features and developmental skills were negatively correlated with later adaptive behaviour. That the association of social autism features with adaptive behaviour was non-significant when controlling for shared variance with developmental skills suggested a *stronger* associative effect of developmental skills with adaptive outcomes. Conversely, the association for developmental skills with adaptive behaviour outcomes remained when social autism features were controlled. We found partial support for our hypothesised moderation effect of developmental skills, with a non-significant overall interaction term but follow-up testing revealing a region of significance whereby social autism features were associated with adaptive behaviour only for children whose developmental skills were assessed as very low for their age.

### Comparison to Existing Literature

The current findings are consistent with those from other research on developmental skills and adaptive behaviour in autism. For instance, among a large sample of > 1,000 young people, Kanne et al. (2010) found no association of global measures of core autism features with concurrent adaptive behaviour, but developmental/cognitive skills (operationalised in terms of IQ) predicting up to 29% of variance in composite adaptive behaviour scores. Similarly, Paul et al. (2011) and Ray-Subramanian et al. (2011) found scores on the adaptive behaviour domain of Daily Living Skills to be better predicted by non-verbal developmental skills than core autism features among autistic toddler-aged samples.

Our results also replicate those of Perry et al. (2009) who found both developmental skills and core autism features to be associated with global adaptive behaviour scores, but did not examine the associated effect of either predictor controlling for shared variance with the other. Flanagan et al. (2015) also found developmental skills to be associated with overall adaptive behaviour, but did not report any direct assessment of association of core autism features with adaptive behaviour. Our formal moderation test demonstrates that developmental skills appear to carry the bulk of shared variance that may explain the association otherwise observed between social autism features and adaptive behaviour outcomes. Hence, the current findings converge broadly with the existing empirical literature assessing both core autism features and developmental skills in relation to adaptive behaviour, while filling a gap by examining these factors simultaneously.

Other published findings appear to contradict the current results. For instance, Bradshaw et al. (2019) found that the adaptive behaviour of autistic toddlers—specifically within the Social and Communication Skill domains—was significantly lower than that of non-autistic toddlers matched on developmental skills, suggesting substantial contribution of core autism features to functional/adaptive disability beyond any association with developmental skills. Further, while Paul et al.’s (2011) primary findings converge with our own, their secondary findings indicated core autism features to be significantly correlated with adaptive behaviour in the Communication Skills domain, in the absence of any link to developmental skills. Plausibly, different patterns of associative influence might emerge from assessment at the subdomain level, distinct from those at the higher-order domain level. For this first examination of potential moderated influence of developmental skills on the association between core autism features and adaptive behaviour outcomes, our decision was to use the global composite score as a proxy for functional ability/impairment—representing overall adaptive skills—rather than focussing on specific domains, which presents an avenue for further exploratory research.

This is the first study, to our knowledge, to test the potential for a specific *moderating* effect of developmental skills on the association between social autism features and adaptive behaviour outcomes. The overall moderation effect approached significance, and we identified a lower-bound region of significance whereby core social autism features were associated with adaptive behaviour specifically for children with *very low* (vs. age-expected) developmental skills. That is, for autistic children with profoundly impacted developmental/learning skills, core social features of their autism are associated with adaptive outcomes one year later, whereas for most autistic children whose developmental skills are moderately/mildly impaired or even at/above age-expected levels, it is these learning skills (rather than core features of their autism) that are more strongly associated with adaptive outcomes.

These results bolster Vivanti et al.’s (2013) assertion that intellectual disability may not simply be an independent feature often *co-occurring* with autism, but rather arise as a function of interacting core features and associated developmental skills that support learning for *all* children. In addition to the unidirectional path from earlier core autism features to later developmental skills demonstrated by Vivanti et al. (2013), and the bidirectional cross-lagged paths we have shown more recently in an expanded cohort (McGowan et al., 2022), here we further expanded this line of investigation to show a link to adaptive behaviour as a broader outcome construct. This is an important extension in the context that adaptive function is increasingly advocated as a desired intervention outcome by the autistic and autism communities (vs. the historically-targeted reduction in core autism features; Gardiner & Iarocci, 2015; Hodge et al., 2021).

### Contribution to Compensation Theory

The moderating effect of developmental skills reported here supports the notion of cognitive compensation over ‘neurotypical’ social learning processes as a primary driver of adaptive outcomes in autism. That is, cognitive compensation theory proposes that an autistic person may implicitly use alternative cognitively-mediated processes to overcome difficulties in otherwise neurotypical cognitive pathways (Livingston & Happé, 2017). In the current data, the observed association between earlier social autism features on later adaptive behaviour moderated by developmental skills suggests the influence of social autism features on practical day-to-day functioning depends to some extent on whether or not developmental skills are also impaired. Specifically, core social autism features may be predictive of adaptive behaviour outcomes in early childhood, only when developmental skills are profoundly *below* age-expected levels; that is, when children cannot employ cognitive skills to compensate for the core social difficulties/differences associated with their neuro-developmental condition.

An interesting inconsistency exists between our findings and those of Liss et al. (2001) who reported that core autism features were negatively associated with concurrent adaptive behaviour among 9-year olds when IQ scores were greater than 80, compared to those with IQ below 80 where there was no significant association between autism and adaptive behaviour measures. While not tested within a moderator model, Liss et al.’s findings imply an ‘upper-bound’ difference (vs. our observed lower-bound region of significance) suggesting that it is children with higher IQ—in line with population average—for whom there is greater impact of core autism features on functional ability. This apparently divergent finding may be a function of participant age/developmental stage; with Liss et al.’s sample 5–6 years older on average than ours. That is, the interactive effects of core autism features and developmental skills on adaptive behaviour outcomes may change with age.

Livingston, Bolton and Happé (2018) demonstrated effects which they interpreted as evidencing compensation—specifically, disparity between observed behaviour and measured underlying skills—among those with higher level IQ and executive functioning scores, in a sample of 136 autistic adolescents, finding an average IQ of 85 among adolescents with lower/no signs of compensation. At face value, these data may suggest that average/above-average IQ is necessary for ‘cognitive compensation’ to occur in autism. Our results, however, suggest the possible manifestation of a ‘cognitive compensation’ of sorts may be observable across autistic children with a broader range of developmental skills (i.e., in all but the most profoundly impaired). Thus, our data from a pre-school aged sample also expands the notion of cognitive compensation as a potentially innate/passive process (Livingston & Happé, 2017) operating much earlier than shown by Livingston et al. (2018).

### Limitations and Future Directions

The current study was limited by characteristics of the sample. All children were engaged with a community service offering the G-ESDM, and presented with sufficiently pronounced difficulties/delays to have been identified early in the community so as to engage with this service. Hence, the current findings may not generalise to samples of young autistic children with fewer early-life difficulties or engaged with different early intervention experience. However, the sample demonstrated wide variability in scores on key measures—spanning the total possible range on assessments of developmental skills and core autism features—as well as in the follow-up interval (which we considered as a proxy for the amount of intervention received; ranging from 3 to 44 months). Neither rate of gain on any measure nor follow-up interval was significantly correlated with adaptive behaviour at outcome, so these variables are unlikely to explain the pattern of results obtained. However there may be factors related to mechanisms of change in the context of G-ESDM—unmeasured here and thereby omitted in the current models—that may influence adaptive behaviour outcomes and the evident moderated effect of core autism features by developmental abilities, and may be elucidated with data from other samples. Future attempts to replicate the current results in other samples, including where supports other than ESDM are received, are therefore warranted to confirm the generalisability of findings to the wider population.

Another limitation is the potential that our analysis was statistically underpowered. While observed power for the overall regression model and detected region of significance was sufficient, a sample of 395 children would be required to detect an interaction term as significant at ≥80% power across the full range of developmental skills as a continuous variable (vs. the present limited lower-bound region of significance, with a non-significant overall interaction term). Again, further replication drawing on data from a larger sample would give increased confidence in the potential for developmental skills to moderate the association between core social autism features and adaptive behaviour outcomes, including potential lower- and upper-bound regions of significance.

Further to achieving a larger sample size, future research should also incorporate longer, multistage follow-ups. Karmiloff-Smith et al.’s (2003) *neuroconstructivist* framework proposed development as a dynamic, contextual process and while the current findings suggest lesser influence of core social autism features than developmental skills on outcomes related to functional ability/disability among preschool aged children, our own past research suggests *bidirectional* influence between core features and developmental skills (McGowan et al., 2022). Moreover, others’ research suggests potentially varying patterns of association among measures at different child ages/developmental stages (e.g., Liss et al., 2001). Future longitudinal studies could seek to identify at what point in development core autism features and developmental skills might be directionally associated with one another and with broader functional ability or disability outcomes.

Finally, and as already outlined, past research examining adaptive behaviour at subdomain level suggests the potential for differential associations with core autism features and/or developmental skills. Important considerations in seeking to further understand directionality of associative effects is to include shared method variance (e.g., strong associations as a function of common parent-report measures across domains/constructs) and item-/domain-level overlap (i.e., adaptive social behaviour encapsulating the social features of autism, rather than viewing these as related but distinct constructs). Future well-powered studies seeking to unravel sub-domain level associations would be welcome, including further consideration of conceptual and measurement-related issues along with the contribution of restricted/repetitive features of autism as potential contributors to adaptive outcomes (e.g. see Glod et al., 2015).

## Declarations

### Credit statement

Conceptualization: Daniel Berends, Catherine Bent, Kristelle Hudry; Methodology: Daniel Berends, Catherine Bent, Kristelle Hudry; Formal analysis and investigation: Daniel Berends; Writing—original draft preparation: Daniel Berends, Kristelle Hudry; Writing—review and editing: Catherine Bent, Giacomo Vivanti, Cheryl Dissanayake; Resources: Catherine Bent, Giacomo Vivanti, Cheryl Dissanayake, Kristelle Hudry; Supervision: Catherine Bent, Kristelle Hudry.
